# Sexual Victimization, Self-Efficacy to Refuse Sex While Drinking, and Regretting Alcohol-Involved Sex among Underserved Youth in Kampala, Uganda

**DOI:** 10.3390/ijerph19041915

**Published:** 2022-02-09

**Authors:** Monica H. Swahn, Rachel E. Culbreth, Amanda K. Gilmore, Dominic J. Parrott, Leah E. Daigle, Rogers Kasirye, Paul Bukuluki

**Affiliations:** 1 Department of Health Promotion and Physical Education, Wellstar College of Health and Human Services, Kennesaw State University, Kennesaw, GA 30144, USA; 2 Department of Respiratory Therapy, Byrdine F. Lewis College of Nursing and Health Professions, Georgia State University, Atlanta, GA 30303, USA; rculbreth@gsu.edu; 3 Center for Research on Interpersonal Violence, Georgia State University, Atlanta, GA 30303, USA; agilmore12@gsu.edu (A.K.G.); parrott@gsu.edu (D.J.P.); ldaigle@gsu.edu (L.E.D.); 4 Department of Health Policy and Behavioral Sciences, School of Public Health, Georgia State University, Atlanta, GA 30303, USA; 5 National Center for Sexual Violence Prevention, School of Public Health, Georgia State University, Atlanta, GA 30303, USA; 6 Department of Psychology, College of Arts and Sciences, Georgia State University, Atlanta, GA 30303, USA; 7 Uganda Youth Development Link, Kampala P.O. Box 12659, Uganda; kasiryer@yahoo.com; 8 College of Humanities and Social Sciences, Makerere University, Kampala P.O. Box 10218, Uganda; pbukuluki@gmail.com

**Keywords:** sexual assault, rape, alcohol use, problem drinking, youth, Africa

## Abstract

The purposes of this study were to determine whether youth who have experienced sexual victimization (SV) have lower self-efficacy to refuse sex and to identify intervention strategies for rape survivors to mitigate further health-risks and harm. Cross-sectional data from the 2014 Kampala Youth Survey (*n* = 1134) of youth aged 12 to 18 years recruited from Uganda Youth Development Link drop-in centers were used to conduct the analyses. Multivariable statistics were computed to determine the correlates (i.e., sex, education, homelessness, problem drinking, and SV) for (1) self-efficacy to refuse sex, (2) self-efficacy to refuse sex while drinking, and (3) regretting sex due to alcohol use. Among participants, 16.9% reported SV (79% were female and 21% were male). In the final adjusted model, self-efficacy to refuse sex while drinking was only associated with homelessness (OR: 0.52; 95% CI: 0.36, 0.74). Previous SV was not associated with lower self-reports of self-efficacy to refuse sex compared to those who had not experienced SV. Additionally, SV was not associated with increased reports of regrets for sex attributed to alcohol use. Alcohol prevention strategies for the most at-risk youth, including homeless youth, are warranted to improve self-efficacy to refuse sex among youth living in the slums of Kampala.

## 1. Introduction

Risky sexual behaviors, including condom-less sex, alcohol-involved sex, and multiple sexual partners, remain a substantial driver of the human immunodeficiency virus (HIV) epidemic in sub-Saharan Africa [1,2,3,4]. Furthermore, risky sexual behaviors are associated with many adverse outcomes, including sexually transmitted diseases (STIs), unwanted pregnancies, and adverse mental health outcomes [5,6]. Risky sexual behaviors are highly prevalent among youth living in the slums of Kampala, Uganda [7], who face substantial adversities, including food scarcity, lack of healthcare infrastructure, and high levels of exposure to violence [8,9,10,11]. This population reports a high level of alcohol use (32%) and a high level of previous sexual victimization (32%) [8], increasing the risk of risky sexual activities and consequentially, adverse mental and physical health effects.

Previous Sexual Victimization (SV) has strongly been linked with subsequent risky sexual behaviors [12,13,14]. Sexual victimization typically refers to any act of coerced sex or forced sex in marriage and dating relationships (intimate partner violence), forced marriage, rape, forced prostitution, unwanted sexual contact, and sexual trafficking [15]. The prevalence of women experiencing any type of intimate partner violence (IPV) in sub-Saharan Africa is among the highest in the world and ranges from 30–66% [16]. Additionally, non-partner SV is also very high in Africa (12%) compared to globally (7%) [17]. In fact, SV in Kampala has been documented as highly prevalent among girls and young women.

In our previous research, we found a high prevalence of IPV (18.3%) [18], rape (16.9%) [19], gender-based violence (GBV) (31.7%) [8], sex work (13.7% of sexually active youth) [20], and commercial sexual exploitation (39.7% of sexually active youth) [21] among youth living in the slums of Kampala. The national Violence Against Children Survey (VACS) in Uganda in 2018 reported that the prevalence of sexual violence victimization prior to age 18 was 35.3% [22]. Additionally, 30% of young women in urban areas of Uganda reported being raped [23]. SV is even higher among at-risk groups, such as young women and girls engaged in sex work. Rape among youth living in the slums of Kampala who were involved in sex work was 67.9% [20].

Several theories may explain the potential link between previous SV and risky sexual behaviors. Emotion dysregulation theory states that SV has a significant impact on emotional regulation, and through emotional dysregulation, individuals experience a decreased ability to adequately control emotional responses, specifically negative emotional states [24,25]. Additionally, emotional dysregulation may lead to impulsivity when experiencing negative emotions [24]. Emotional numbing (separating emotions from current experiences) has been associated with reduced self-efficacy among young women with previous SV who consume alcohol [26]. Traumagenics theory may also explain the association between SV and risky sexual behaviors [12,27]. Traumatic sexualization in childhood may be associated with experiences of childhood rewards and affection, consequently leading to a dysfunctional relationship between sexual relations in adulthood and expectations of receiving rewards and affection [12,27].

Self-efficacy to refuse sex is a strong protective factor against unwanted sexual activity and is a modifiable mediator between prior SV and risky sexual behaviors [28]. Self-efficacy to refuse sex includes maintaining the confidence and intention to refuse unwanted sexual encounters [29,30,31,32]. SV and self-efficacy to refuse sex are frequently complicated by concurrent substance use, including alcohol use [33]. Alcohol use is known to reduce self-efficacy to refuse sex through alcohol’s disinhibition effects, impulsivity characteristics, and altered decision-making capacity [4,15]. In Uganda, the prevalence of heavy episodic drinking among women is 7.8% [34]. However, among drinkers, the prevalence of heavy episodic alcohol use is 32.6%. Even among youth, alcohol use prevalence is estimated at 30% for youth in Kampala [9]. GBV, and more specifically, SV have been consistently linked to alcohol use, and there is extensive literature on the extent to which involving alcohol contributes to violence victimization. In fact, an estimated one-third of SV episodes involve alcohol use [35]. Alcohol use is thought to have a disinhibitory effect [35], potentially explaining the link between alcohol use and GBV and other forms of violence through intense aggression and impaired judgment for both perpetrator and victim. Additionally, polysubstance use has also been shown to lower serotonin levels, further increasing aggression in GBV perpetrators and potentially impairing judgment in GBV victims [35,36]. Other drugs are often used to cope with the pain experienced from GBV episodes, which may further increase risk of re-victimization [15,37]. Unfortunately, the concurrence of alcohol use and SV (factoring in both victimization and perpetration) is highly prevalent among youth in the slums of Kampala. GBV and alcohol use rarely exist in isolation in this population as reported in our previous research [8].

Self-efficacy to refuse sex, particularly while drinking, is a critical factor in building resiliency among youth and protecting youth from the adverse consequences of SV and STIs, including HIV. Additionally, youth who report regretting alcohol-involved sex may also be key targets that may benefit from secondary prevention programs. However, based on previous research indicating that SV may lead to reduced self-efficacy, it is critical to empirically examine this specific link in these underserved populations with high levels of violence victimization. Thus, the research questions that informed this study were as follows: What is the prevalence of self-efficacy to refuse sex and self-efficacy to refuse sex while drinking?; Are the levels of self-efficacy to reduce sex different for those having experienced SV?; and what is the prevalence of regretting alcohol-involved sex, and is this different for those having experienced SV? We hypothesize that youth who reported a previous SV episode will have significantly lower prevalence of self-reported efficacy to refuse sex compared to youth who did not report a previous SV episode. We also hypothesize that youth who reported a previous SV episode will have significantly higher prevalence of regretting alcohol-involved sex compared to youth who did not report a previous SV episode. Our final hypothesis is that problem drinking would serve as a moderator for the association between SV and self-efficacy to refuse sex, such that SV and self-efficacy to refuse sex would exhibit a positive, stronger association among problem drinkers compared to non-drinkers and non-problem drinkers. These hypotheses are primarily driven by previous research indicating less self-efficacy following SV among youth who drink [23]. The results from this study intend to inform the development of effective and highly scalable primary and secondary prevention interventions for alcohol-involved sex and SV in this vulnerable and underserved population.

## 2. Materials and Methods

### 2.1. Study Sample

The current study consists of a convenience sample of youth (*n* = 1134) aged 12–18 who were living in the slums of Kampala in 2014 and attended drop-in centers at Uganda Youth Development Link (UYDEL). UYDEL provides services such as counselling, STI/HIV testing, vocational training, and linkages to other care. Further details of this study and questionnaire are documented elsewhere [8,9,20].

### 2.2. Measures

The main independent variable of interest was sexual victimization (SV) and was operationalized using the question, “Has someone ever raped you or forced you to have sex with him or her?” The three outcomes of this study were self-efficacy to refuse sex, self-efficacy to refuse sex while drinking, and regretting sex due to alcohol. Self-efficacy to refuse sex was operationalized as agreeing, disagreeing, or neither agreeing nor disagreeing to the statement, “I would be able to refuse to have sex with my boyfriend/girlfriend if I did not feel like having sex.” Self-efficacy to refuse sex while drinking was operationalized as agreeing, disagreeing, or neither agreeing nor disagreeing to the statement, “I would be able to refuse to have sexual intercourse even if I had been drinking alcohol.” Regretting sex due to alcohol use was operationalized using the question, “Because of your own alcohol use, how often during the last 12 months have you experienced the following: had sex which you wished you hadn’t the next day?” Participants could answer “0 times or never,” “1–2 times,” and “3–4 times.” Additionally, this question was only asked among drinkers.

Other independent variables included homelessness (“Have you ever lived on the streets with nowhere else to go?”), education (completed less than primary education, completed primary education, or some secondary education or higher), sex (male/female), age, and problem drinking. Problem drinking was assessed via the four-item Cut-Annoyed-Guilty-Eye-opener (CAGE) questionnaire: (1) “Have you ever felt you should cut down on your drinking?”; (2) “Have you ever had people annoy you by criticizing your drinking?”; (3) “Have you ever felt bad or guilty about your drinking?” and (4) “Have you ever had a drink first thing in the morning to steady your nerves or get rid of a hangover (eye-opener)?” Problem drinking was computed by adding responses to each question (responses of “Yes” were given 1 point). Individuals who totaled 2 or more points were classified as having engaged in problem drinking (coded as 2). For those who totaled 1 or 0 points, they were classified as having engaged in non-problem drinking (coded as 1). For non-drinkers, they were classified as simply “non-drinkers” (coded as 0, or reference group).

### 2.3. Data Analysis

Descriptive statistics were computed for all variables, and binary and multinomial logistic regression analyses were conducted for the outcomes of self-efficacy to refuse sex (disagree/neither agree nor disagree/agree), self-efficacy to refuse sex while drinking (disagree/neither agree nor disagree/agree) and regretting sex due to alcohol use (0 times, 1–2 times, 3–4 times). Predictors included sex, education, homelessness, problem drinking, and SV. The moderating effect of problem drinking on the association between SV and self-efficacy to refuse sex was also assessed.

## 3. Results

Nearly 17% of participants reported a history of SV victimization. Among females, 23.7% reported being raped, while 8.1% of males reported experiencing SV victimization. Those with higher education also had a higher prevalence of reporting previous SV compared to those who had not completed primary school. Youth who reported a history of homelessness had more than double the prevalence of SV compared to youth who did not report a history of homeless (29.4% vs. 13.3%, respectively). Additionally, those who reported alcohol use in the past year had much higher prevalence of SV (27.8%) compared to those who reported not drinking alcohol in the past year (12.1%).

Table 1 presents characteristics of self-efficacy to refuse sex among youth living in the slums of Kampala. Overall, the majority of youth (83.3%) reported self-efficacy to refuse sex. Approximately 7% of males reported not having self-efficacy to refuse sex, which was higher than the percentage for females (4%) ($\chi^{2}$ = 8.67, df = 2, *p* = 0.01). Those ever homeless had a slightly higher percentage of lacking self-efficacy to refuse sex (9.2%) compared to those never homeless (4.4%). This difference was statistically significant ($\chi^{2}$ = 11.45, df = 2, *p* = 0.03). Lacking self-efficacy to refuse sex was also much higher among problem drinkers (10.4%) compared to non-problem drinkers (6.1%) and non-drinkers (4.3%) ($\chi^{2}$ = 13.29, df = 4, *p* < 0.01). Self-efficacy to refuse sex was associated with being female in the multivariable model (OR: 1.92; 95% CI: 1.08, 3.41), while problem drinking was associated with a reduced odds of self-efficacy to refuse sex (OR: 0.41; 95% CI: 0.21, 0.81). The moderating effect of problem drinking on the association between rape and self-efficacy to refuse sex was not statistically significant.

Characteristics of self-efficacy to refuse sex while drinking are presented in Table 2. Overall, a higher percentage of youth reported no self-efficacy to refuse sex while drinking (22.3%) compared to youth who reported no self-efficacy to refuse sex overall (12.2%). The percentages of youth who reported no self-efficacy to refuse sex while drinking were similar for both males and females (22.5% vs. 22.1%, respectively) and across education levels. Those with a history of homelessness had a much higher self-reported lack of self-efficacy to refuse sex while drinking (31.6%) compared to those never homeless (19.6%) ($\chi^{2}$ = 15.92, df = 2, *p* < 0.01). Additionally, those categorized as problem drinkers reported a higher prevalence of no self-efficacy to refuse sex while drinking (27.4%) compared to non-problem drinkers (23.3%) and non-drinkers (20.9%) ($\chi^{2}$ = 11.24, df = 4, *p* = 0.02). Finally, those who reported experiencing SV had a higher prevalence of no self-efficacy to refuse sex while drinking (26.2%) compared to those who have not reported previous SV (21.5%). However, this association was not statistically significant ($\chi^{2}$ = 5.80, df = 2, *p* = 0.05). Predictors of self-efficacy to refuse sex while drinking are presented in Table 2. In the multivariable model, homelessness was the only variable that retained a significant association, with a reduced odds of self-efficacy to refuse sex while drinking (OR: 0.52, 95% CI: 0.36, 0.74).

Descriptive statistics of youth who reported having sex that they regretted the next day due to alcohol use are presented in Table 3. A higher percentage of females (16.2%) reported having sex that they regretted due to alcohol use three or more times compared to males (9.0%). Youth with a history of homelessness had a much higher prevalence (19.4%) of having sex three or more times that they regretted due to alcohol use compared to non-homeless individuals (8.9%). Similar patterns were observed for problem drinkers and those experiencing previous SV, indicating higher prevalence of regretting sex due to alcohol. Table 3 also presents the bivariate and multivariable analyses for sex that youth regretted due to alcohol use. In the multivariable analyses, homelessness (OR: 2.09; 95% CI: 1.03, 4.23) and problem drinking (OR: 2.02; 95% CI: 1.02, 4.00) were associated with having sex three or more times that the youth regretted due to alcohol.

An analysis was also computed to determine the proportion of those who reported self-efficacy to refuse sex while drinking among the percentage of youth who reported regretting sex due to alcohol. In this analysis limited to only drinkers, 60% of youth reported self-efficacy to refuse sex while drinking. Among those, 45% reported at least one incident of regretting sex due to drinking.

## 4. Discussion

This study empirically examined levels of self-efficacy to refuse sex and regretting engaging in alcohol-involved sex by key demographic characteristics and SV in a high-risk population in Uganda. This population has been found to have high levels of violence and alcohol in other previous studies [8,10,18,19]. Our hypotheses, consistent with previous research [26], were that self-efficacy to refuse sex, as well as self-efficacy to refuse sex while drinking, would be lower among SV survivors compared to those who had not experienced SV. Contrary to our hypotheses, we did not find an association between SV and reduced self-efficacy to refuse sex nor reduced self-efficacy to refuse sex while drinking. Additionally, we did not find an association between SV and regretting sex due to alcohol use. This highlights a key finding in that other factors may play a larger role in self-efficacy to refuse sex above and beyond sexual victimization. However, in the descriptive analyses, youth who had experienced SV comprised a larger percentage of those reporting reduced self-efficacy to refuse sex compared to those who had not experienced SV. Therefore, while it is clear that youth who experienced SV had a higher percentage of reporting reduced self-efficacy to refuse sex, other factors may be more important in youth’s reduced self-efficacy to refuse sex.

Failing to detect an association between SV and self-efficacy to refuse sex is contradicted by previous literature [33,38,39]. While SV may play a role in reduced self-efficacy to refuse sex in certain populations, future studies should examine the interrelationships between SV and other variables in this population. Youth living in the slums of Kampala may be fundamentally different from other populations with regards to the association between SV and reduced self-efficacy to refuse sex.

In the third set of analyses, we examined incidents of regretting sex due to drinking. We hypothesized that SV survivors would have a higher prevalence of regretting sex due to alcohol for the same reason that we had also hypothesized that they would have less self-efficacy to refuse sex. Although the latter hypotheses were not confirmed, SV survivors did indeed have higher prevalence of sexual incidents that they regretted due to alcohol. These incidents were most commonly reported by females, those reporting a history of homelessness, problem drinkers, and those previously reported SV.

Intriguingly, among those who reported self-efficacy to refuse sex while drinking, nearly half reported at least one incident of regretting sex due to drinking. Perhaps these inconsistencies can be best understood in the context of expected versus actual behaviors [40]. When answering the questions about self-efficacy to refuse sex, a hypothetical scenario perhaps, the majority of youth believed they could refuse sex (88%), and more than half believed they could refuse sex while drinking (57%). However, the fact that nearly half of those who believed they could refuse sex while drinking also reported that they regretted incidents of sex due to their drinking, underscore the challenges of self-efficacy to refuse sex while under the influence. These findings, however, are consistent with previous research on alcohol use and reduced self-efficacy to refuse sex [26,38].

Females also were more likely to report self-efficacy to refuse sex without specific reference to alcohol involvement. This finding is similar to another study among adolescents in South Africa, where female adolescent youth reported a higher self-efficacy to refuse sex compared to male adolescent youth [41]. In the aforementioned study, females were more aware of HIV prevention campaigns and safe sexual practices compared to males, which may explain the gender differences in self-efficacy [41]. Additionally, in our study, we did not find an association between females and self-efficacy to refuse sex with alcohol involvement. Percentages were nearly identical for males and females for reporting no self-efficacy to refuse sex while drinking. These high percentages of reduced self-efficacy to refuse sex for both males and females represent an important finding for alcohol prevention interventions. Females were less likely to drink compared to males in our previous research among youth living in the slums of Kampala [9], but research also indicates that females experience a higher burden of alcohol-related adverse effects compared to males [42], even when drinking the same amount. Future research should investigate these sex-related differences between alcohol-related self-efficacy for sex refusal in this population and potential age-related modifying effects that could serve as intervention points. Additionally, these findings represent a need to develop gender-specific messages for self-efficacy to refuse sex, since the drivers of self-efficacy to refuse sex may be different by gender.

Youth who reported problem drinking were more likely to report lower self-efficacy to refuse sex. However, problem drinking was not a statistically significant moderator of the association between SV and self-efficacy to refuse sex while drinking, nor between SV and self-efficacy to refuse sex more broadly. However, it is clear that a higher percentage of youth reported the inability to refuse sex while drinking compared to the inability to refuse sex more broadly. This carries potentially significant intervention implications for this population, where alcohol use is highly prevalent and known to have a wide range of adverse outcomes. It is also clear that youth are overly confident in their ability to refuse sex while drinking as indicated by the high proportion, nearly half, who report regretting incidents of sex due to alcohol. Problem drinking was also a statistically significant predictor of overall self-efficacy to refuse sex more broadly, underscoring the potential influence of alcohol on reduced self-efficacy to refuse sex in this population. Future research should examine effective alcohol prevention interventions in the context of sexual risk behaviors for these youth, who do not have adequate access to healthcare infrastructure and government services.

In analyses of self-efficacy to refuse sex while drinking, the key correlate was homelessness, which was associated with less self-efficacy to refuse sex while drinking. One potential underlying explanation for this association may be survival sex, with the youth reporting reduced self-efficacy for refusal because the youth may be receiving shelter, food, or money in exchange for sexual encounters [20,21,43]. Sex work was not assessed in these analyses, but our previous research demonstrated that youth involved in sex work experience a much higher range of adverse sexual consequences, including rape, compared to youth who are not involved in sex work [20].

While we did not detect an association between SV victimization and reduced self-efficacy to refuse sex, a higher percentage of SV survivors reported reduced self-efficacy to refuse sex while drinking compared to those who did not experience SV. In the bivariate logistic regression analyses, youth were more likely to report not having self-efficacy to refuse sex while drinking compared to responding, “Neither agree nor disagree.” While this was association was not statistically significant in the multivariable model, this still represents a potential intervention target for individuals who have experienced SV. This finding is also consistent with previous research, which suggests that alcohol is associated with emotional numbing and reduced self-efficacy to refuse sex among young women with previous childhood sexual abuse [26]. Sexual assault emergency resources are rare for young women living in the slums of Kampala [44], and culturally appropriate interventions are needed for these women to reduce the sequelae of SV.

### Limitations

While this study presents important findings related to self-efficacy to refuse sex among a high priority and underserved population, there are several limitations, particularly as we conducted secondary analyses on a topic not originally intended for the dataset. First, this cross-sectional study cannot infer causality, and we did not assess these relationships longitudinally. Next, we did not assess the timeframe for the rape experiences and homelessness. Therefore, we are unsure how timing of these factors may impact the outcomes and associations. Youth who attended UYDEL may be more health conscious than youth in the general population of Kampala since these youth are service-seeking. However, we are unsure how these findings generalize to other youth in Kampala. Despite these limitations, our findings raise important questions for future research and concerns about youth confidence in refusing sex and the influence of alcohol on decision making in a fairly large sample of high-risk youth.

## 5. Conclusions

Alcohol use continues to be one of the main drivers for risky sexual behaviors among youth living in the slums of Kampala. This study found that youth with a history of homelessness were less likely to report self-efficacy to refuse sex while drinking, likely driven by survival sex. We did not find an association between SV and reduced self-efficacy to refuse sex. It is also clear from this study that youth are overly confident in their ability to refuse sex while drinking as indicated by the high proportion, nearly half, who reported regretting incidents of sex due to alcohol. Future studies should be conducted to better understand the contextual factors that increase risk for alcohol-involved sex that may be unplanned and later regretted in order to increase skills and self-efficacy to reduce health risks and harm. Future research should also examine a larger sample across more diverse study sites to determine more generalizable drivers for self-efficacy to refuse sex. It is clear that culturally appropriate, scalable, and feasible interventions that can be implemented in low-resource settings are urgently needed to address high-risk sexual behaviors and alcohol use in this and similar underserved populations.

## Figures and Tables

**Table 1:** Prevalence and Correlates of Self-efficacy to Refuse Sex among Youth Living in the Slums of Kampala, Uganda (*n*= 1134).

Independent Variables	Self-Efficacy to Refuse Sex—No *n* = 62 (5.5%)	Self-Efficacy to Refuse Sex—Neither Agree/Disagree *n* = 127 (11.2%)	Self-Efficacy to Refuse Sex—Yes *n* = 945 (83.3%)	Total *n* = 1134	Logistic Regression Analyses of Self-Efficacy to Refuse Sex			
					Unadjusted		Adjusted	
					Neither Agree Nor Disagree	Agree-Self-Efficacy to Refuse Sex while Drinking	Neither Agree nor Disagree	Agree-Self-Efficacy to Refuse Sex while Drinking
Sexual victimization								
No	46 (4.9%)	107 (11.4%)	786 (83.7%)	939 (83.1%)	1.00	1.00	1.00	1.00
Yes	13 (6.8%)	19 (10.0%)	159 (83.3%)	191 (16.9%)	0.63 (0.29, 1.38)	0.72 (0.38, 1.36)	0.66 (0.28, 1.57)	0.72 (0.35, 1.46)
Sex *								
Male	37 (7.4%)	62 (12.5%)	398 (80.1%)	497 (43.9%)	1.00	1.00	1.00	1.00
Female	25 (3.9%)	65 (10.2%)	546 (85.9%)	636 (56.1%)	1.55 (0.84, 2.87)	**2.03 (1.20, 3.43)**	1.50 (0.77, 2.93)	**1.92 (1.08, 3.41)**
Education *								
Less than primary	25 (6.3%)	66 (16.6%)	306 (77.1%)	397 (35.5%)	1.00	1.00	1.00	1.00
Completed primary	12 (4.6%)	21 (8.0%)	230 (87.5%)	263 (23.5%)	0.66 (0.29, 1.54)	1.57 (0.77, 3.18)	0.65 (0.28, 1.53)	1.47 (0.72, 3.02)
Secondary or more	24 (5.2%)	36 (7.8%)	400 (87.0%)	460 (41.1%)	0.57 (0.28, 1.14)	1.36 (0.76, 2.43)	0.66 (0.32, 1.35)	1.55 (0.84, 2.87)
Homelessness *								
No	39 (4.4%)	93 (10.5%)	752 (85.1%)	884 (78.0%)	1.00	1.00	1.00	1.00
Yes	23 (9.2%)	34 (13.7%)	192 (77.1%)	249 (22.0%)	0.62 (0.32, 1.19)	**0.43 (0.25, 0.74)**	0.75 (0.36, 1.54)	0.66 (0.36, 1.20)
Problem drinking *								
Non-drinker	34 (4.3%)	82 (10.4%)	674 (85.3%)	790 (69.7%)	1.00	1.00	1.00	1.00
Non-problem drinking	11 (6.1%)	21 (11.7%)	148 (82.2%)	180 (15.9%)	0.79 (0.35, 1.82)	0.68 (0.34, 1.37)	0.88 (0.37, 2.08)	0.73 (0.35, 1.51)
Problem drinking	17 (10.4%)	24 (14.6%)	123 (75.0%)	164 (14.5%)	0.59 (0.28, 1.23)	**0.37 (0.20, 0.67)**	0.70 (0.31, 1.55)	**0.41 (0.21, 0.81)**

Note. Reference group = disagree/no self-efficacy to refuse sex while drinking. Moderator of problem drinking for association between SV and self-efficacy of sex while drinking not statistically significant. * = *p*-value < 0.05 for chi-square tests between self-efficacy to refuse sex and independent variables. Statistically significant associations for the logistic regression analyses are bolded.

**Table 2:** Prevalence and Correlates of Self-efficacy to Refuse Sex while Drinking among Youth Living in the Slums of Kampala, Uganda (*n* = 1134).

Independent Variables	Self-Efficacy to Refuse Sex while Drinking—No *n* = 251 (22.3%)	Self-Efficacy To Refuse Sex while Drinking—Neither Agree/Disagree *n* = 235 (20.8%)	Self-Efficacy to Refuse Sex while Drinking—Yes *n* = 642 (56.9%)	Total *n* = 1128	Logistic Regression Analyses of Self-Efficacy to Refuse Sex while Drinking			
					Unadjusted		Adjusted	
					Neither Agree nor Disagree	Agree-Self-Efficacy to Refuse Sex while Drinking	Neither Agree nor Disagree	Agree-Self-Efficacy to Refuse Sex while Drinking
Sexual victimization								
No	201 (21.5%)	206 (22.0%)	530 (56.6%)	937 (83.1%)	1.00	1.00	1.00	1.00
Yes	50 (26.2%)	28 (14.7%)	113 (59.2%)	191 (16.9%)	**0.55 (0.33, 0.91)**	0.87 (0.60, 1.27)	0.68 (0.40, 1.17)	1.01 (0.67, 1.51)
Sex								
Male	111 (22.5%)	98 (19.9%)	284 (57.6%)	493 (43.7%)	1.00	1.00	1.00	1.00
Female	140 (22.1%)	137 (21.6%)	358 (56.4%)	635 (56.3%)	1.12 (0.88, 1.61)	1.00 (0.74, 1.34)	1.12 (0.77, 1.64)	0.94 (0.69, 1.28)
Education *								
Less than primary	83 (21.0%)	105 (26.5%)	208 (52.5%)	396 (35.5%)	1.00	1.00	1.00	1.00
Completed primary	137 (22.5%)	99 (16.3%)	372 (61.2%)	608 (54.5%)	**0.57 (0.39, 0.84)**	1.08 (0.79, 1.49)	0.55 (0.37, 0.82)	1.00 (0.72, 1.39)
Secondary or more	29 (26.1%)	25 (22.5%)	57 (51.4%)	111 (10.0%)	0.68 (0.37, 1.25)	0.78 (0.47, 1.31)	0.64 (0.34, 1.18)	0.72 (0.43, 1.22)
Homelessness *								
No	173 (19.6%)	189 (21.5%)	519 (58.9%)	881 (78.1%)	1.00	1.00	1.00	1.00
Yes	78 (31.6%)	46 (18.6%)	123 (49.8%)	247 (21.9%)	**0.53 (0.35, 0.81)**	**0.53 (0.38, 0.74)**	**0.60 (0.38, 0.95)**	**0.52 (0.36, 0.74)**
Problem drinking *								
Non-drinker	164 (20.9%)	183 (23.3%)	438 (55.8%)	785 (69.5%)	1.00	1.00	1.00	1.00
Non-problem drinking	42 (23.3%)	28 (15.6%)	110 (61.1%)	180 (15.9%)	0.59 (0.35, 1.00)	0.98 (0.66, 1.46)	0.70 (0.40, 1.20)	1.17 (0.74, 1.69)
Problem drinking	45 (27.4%)	24 (14.6%)	95 (57.9%)	164 (14.5%)	**0.50 (0.29, 0.86)**	0.81 (0.55, 1.21)	0.62 (0.35, 1.08)	0.97 (0.64, 1.48)

Note. Reference group = disagree/no self-efficacy to refuse sex while drinking. * = *p*-value < 0.05 for chi-square tests between self-efficacy to refuse sex while drinking and independent variables. Moderator of problem drinking for association between SV and self-efficacy of sex while drinking not statistically significant. Statistically significant associations for the logistic regression analyses are bolded.

**Table 3:** Characteristics of Regretting Sex due to Drinking among Current Youth Drinkers Living in the Slums of Kampala.

Independent Variables	Never	1–2 Times	3 or More Times	Total *n* = 347	Logistic Regression Analyses of Regretting Sex due to Alcohol			
					Unadjusted Model		Adjusted Model	
					1–2 Times	3 or More Times	1–2 Times	3 or More Times
Sexual victimization								
No	140 (55.8%)	84 (33.5%)	27 (10.8%)	251 (72.3%)	1.00	1.00	1.00	1.00
Yes	40 (41.7%)	38 (39.6%)	18 (18.8%)	96 (27.7%)	1.49 (0.88, 2.52)	**2.32 (1.16, 4.63)**	1.36 (0.77, 2.41)	1.41 (0.65, 3.06)
Sex								
Male	93 (60.0%)	48 (31.0%)	14 (9.0%)	155 (44.7%)	1.00	1.00	1.00	1.00
Female	87 (45.3%)	74 (38.5%)	31 (16.2%)	192 (55.3%)	1.59 (0.99, 2.53)	**2.34 (1.17, 4.70)**	1.41 (0.85, 2.32)	2.10 (0.98, 4.50)
Education								
Less than primary	63 (51.2%)	41 (33.3%)	19 (15.5%)	123 (35.8%)	1.00	1.00	1.00	1.00
Completed primary	37 (49.3%)	29 (38.7%)	9 (12.0%)	75 (21.8%)	1.20 (0.64, 2.25)	0.81 (0.33, 1.97)	1.19 (0.63, 2.25)	1.00 (0.40, 2.52)
Secondary or more	79 (54.1%)	50 (34.3%)	17 (11.6%)	146 (42.4%)	0.97 (0.57, 1.65)	0.71 (0.34, 1.49)	0.90 (0.52, 1.55)	0.78 (0.36, 1.69)
Homelessness								
No	111 (52.1%)	83 (39.0%)	19 (8.9%)	213 (61.4%)	1.00	1.00	1.00	1.00
Yes	59 (51.5%)	39 (29.1%)	26 (19.4%)	134 (38.6%)	0.73 (0.45, 1.19)	**2.23 (1.15, 4.34)**	**0.70 (0.42, 1.18)**	**2.09 (1.03, 4.23)**
Problem drinking								
Non-problem drinking	101 (56.1%)	62 (34.4%)	17 (9.4%)	180 (51.9%)	1.00	1.00	1.00	1.00
Problem drinking	77 (47.0%)	59 (36.0%)	28 (17.1%)	164 (47.3%)	1.30 (0.83, 2.04)	**2.24 (1.16, 4.34)**	1.28 (0.81, 2.04)	**2.02 (1.02, 4.00)**

Note. Reference group = 0 times/Never regretted sex due to drinking. Statistically significant associations for the logistic regression analyses are bolded.

## Data Availability

The data presented in this study are available on request from the corresponding author. The data are not publicly available due to ongoing preparation of grants and publications.
